# Cigarette smoke exposure impairs lipid metabolism by decreasing low-density lipoprotein receptor expression in hepatocytes

**DOI:** 10.1186/s12944-020-01276-w

**Published:** 2020-05-08

**Authors:** Baitao Ma, Yunfei Chen, Xuebin Wang, Rui Zhang, Shuai Niu, Leng Ni, Xiao Di, Qin Han, Changwei Liu

**Affiliations:** 1 Department of Vascular Surgery, Peking Union Medical College Hospital, Chinese Academy of Medical Sciences and Peking Union Medical College, Beijing, 100730 China; 2 Medical Science Research Center, Chinese Academy of Medical Sciences and Peking Union Medical College, Beijing, 100730 China

**Keywords:** Cigarette smoke, Lipid, Low-density lipoprotein receptor, Atherosclerosis, Melatonin, Mice

## Abstract

**Background:**

Cigarette smoke (CS) exposure impairs serum lipid profiles and the function of vascular endothelial cells, which accelerates the atherosclerosis. However, the precise mechanism and effect on the expression of low-density lipoprotein receptor (LDLR) in the liver by CS exposure is still unclear.

**Methods:**

In this study, adult male C57BL/6 J mice were divided into three groups, with one group being exposed to CS for 6 weeks. HepG2 cells were treated with CS extract at concentrations of 1, 2.5, 5, and 10%.

**Results:**

The serum levels of total cholesterol (TC), triglycerides (TGs), and low-density lipoprotein cholesterol (LDL-C) for the CS-exposure group were significantly higher than those in the control group (*P* < 0.05). Moreover, CS exposure decreased the LDLR expression in the hepatocytes and promoted inflammation in the blood vessel walls. Melatonin was intraperitoneally injected at 10 mg/kg/d for 6 weeks alongside CS exposure, and this significantly decreased the levels of TC, TGs, and LDL-C and decreased the expression of intercellular adhesion molecule-1 and the infiltration of cluster determinant 68-cells. In vitro, CS extract prepared by bubbling CS through phosphate-buffered saline decreased the LDLR expression in HepG2 cells in a time- and concentration-dependent manner, and this effect was prevented by pretreatment with 100 μM melatonin.

**Conclusions:**

In conclusion, CS exposure impaired lipid metabolism and decreased LDLR expression in hepatocytes, and these effects could be prevented by melatonin supplementation. These findings implied that melatonin has the potential therapeutic applicability in the prevention of lipid metabolic disorder in smokers.

## Background

Atherosclerosis is a chronic inflammatory disease characterized by the accumulation of lipids and fibrous elements in the arteries [1, 2]. Accordingly, atherosclerosis is one of the major causes of cardiovascular disease and a serious threat to human health [3]. Epidemiological studies have demonstrated that cigarette smoke (CS) is a high-risk factor for the development of atherosclerosis, as well as coronary and peripheral vascular disease [4, 5]. Disorder of lipid metabolism caused by CS exposure, particularly elevated low-density lipoprotein cholesterol (LDL-C), are some of the most important atherosclerosis accelerating factors [2, 6]. However, the specific mechanism by which CS perturbs lipid metabolism remains unclear.

Numerous studies on the effects of CS on blood lipids have been performed using different animals and CS-exposure techniques. For example, apolipoprotein E deficient (ApoE−/−) mice fed a high-fat diet and exposed to CS for 15 weeks exhibited changes in vascular lipid profile and significantly accelerated the formation of atherosclerotic plaques, but no statistically significant effect on blood lipid levels was observed. However, in the same study, low-density lipoprotein receptor (LDLR)-deficient (LDLR−/−) mice exposed to CS exhibited significantly increased serum cholesterol levels [7]. In a related study, ApoE−/− mice were fed a normal laboratory diet and exposed to fresh air (control) or CS for three or 6 months. While the CS-exposed mice showed accelerated plaque growth and higher aortic arch cholesterol content, the high-density lipoprotein cholesterol (HDL-C) levels did not differ significantly between the groups [8]. In addition, human cholesteryl ester transfer protein transgenic mice and obese rats also exhibited lipid metabolic disorder after CS exposure [9, 10].

Melatonin (*N*-acetyl-5-methoxytryptamine) is an endogenous indoleamine that is mainly secreted by the pineal gland and has a remarkable range of physiological functions and effects, such as circadian rhythm regulation [11], anti-atherosclerosis [12], anti-inflammation [13], anti-oxidation [14], and immune regulation [15]. Two separate meta-analysis studies have shown that melatonin supplementation could reduce the level of triglycerides (TGs), but its effects on LDL-C are inconsistent [16, 17]. Koziróg et al. demonstrated that patients with metabolic syndrome who received melatonin (5 mg/day) for 2 months showed a significant reduction in LDL-C [18]. However, the molecular mechanism by which melatonin acts on the LDL-C level is currently unknown.

In the present study, male C57BL/6 J mice were used to investigate the in vivo effect of melatonin on dyslipidemia caused by CS. This study also investigated whether the expression of LDLR in HepG2 cells was changed after treatment with cigarette smoke extract (CSE).

## Materials and methods

### Animals and experimental design

Twenty-four special pathogen free (SPF) adult male C57BL/6 J mice weighing between 25 and 30 g were provided by the Laboratory Animal Center of Peking Union Medical College Hospital (PUMCH) and fed a standard laboratory diet containing 0.003% cholesterol and 4.0% fat. The mice were housed in SPF conditions at 25 ± 2 °C and 60% ± 5% humidity under a 12 h light/dark cycle and allowed free access to water and food. All experimental animals were fed adaptively for 2 weeks before initiating the experiment. Mice were divided into three groups (*n* = 8 per group). Group I (sham group) were exposed to fresh air and injected with saline intraperitoneally; Group II (CS group) were exposed to CS and injected with saline intraperitoneally; and Group III (CS + melatonin group) were exposed to CS and injected with melatonin (10 mg/kg/d) (Sigma-Aldrich, M5250) intraperitoneally.

The smoke-exposure equipment was as previously described [19], and comprised a relatively independent glass chamber for observing the behavior of experimental animals during the study. Groups II and III were exposed to the smoke of twenty commercial cigarettes (10 mg tar and 0.8 mg nicotine per cigarette) each day according to the study by Hautamaki et al. [20] with some modifications. The period of daily smoke exposure was 180 min (10 min smoking with 5 min breaks) between 10:00 and 11:30 and between 16:00 and 17:30 for 6 weeks (3 h/day, 6 days/week). Group III were injected intraperitoneally with melatonin at 10 mg/kg/d, while Groups I and II received injections of an equivalent volume of saline.

The body weights of the mice were recorded at 8:00 every Friday. After 6 weeks, the mice were anesthetized by injection of pentobarbital sodium. The collected fresh blood of mice was centrifuged for 20 min (1000 g, 4 °C) after standing for 2 h at room temperature and stored at − 80 °C prior to further biochemical analyses. After removing the surrounding tissues, the thoracic aortas were fixed in 4% paraformaldehyde for immunohistochemical (IHC) assay. The livers were kept in liquid nitrogen for subsequent analyses. All animal experiments were approved by the Institutional Animal Care and Use Committee at PUMCH.

### IHC staining

The IHC staining procedure was carried out as described previously [19, 21]. The thoracic artery was processed using standard procedures in a series of graded alcohols and xylene and then paraffin-embedded. Paraffin-embedded slices were serially sectioned at 5-mm intervals. Then, IHC analyses for intercellular adhesion molecule-1 (ICAM-1) and cluster determinant 68 (CD68) were performed. After being blocked in 5% bovine serum albumin for 30 min at room temperature, the sections were incubated overnight at 4 °C with primary antibodies (1:100; Servicebio, Wuhan, China). The slides were then washed with phosphate-buffered saline (PBS) (3 × 5 min) followed by incubation with horseradish peroxidase-conjugated anti-rabbit IgG (1:5000; Zhongshan Jinqiao Biotechnology, Beijing, China) at room temperature for 1 h. Peroxidase activity was detected by treatment with diaminobenzidine. The stained slides were observed under a microscope (DMI 4000 B; Leica Microsystems) and the expression levels of ICAM-1 and CD68 were quantified as average integrated optical density (IOD) with Image J software.

### Serum lipid analysis

Serum levels of TC, TGs, LDL-C, and HDL-C were determined by using an automatic biochemical analyzer (Rayto, Shenzhen, China) following the manufacturer’s instructions.

### Cell culture and treatment

HepG2 cells were obtained from Dr. Chunling Xue. Cells were propagated in Dulbecco’s modified Eagle’s medium (DMEM) (Hyclone, Logan, UT, USA) supplemented with 10% fetal bovine serum (FBS) (Hyclone, Logan, UT, USA) and 1% (*v/v*) penicillin/streptomycin at 37 °C under 5% CO_2_. For the CSE and/or melatonin treatment experiments, culture medium was changed to 1% FBS-containing DMEM. Cells were stimulated with CSE at a concentration of 5% for 24 h or pretreated with 100 μM melatonin for 2 h. Unless otherwise indicated, all experiments were performed in triplicate and representative results were shown.

### Preparation of CSE

The preparation of CSE was performed as described previously [22, 23]. The CSE was stored at − 80 °C and used within 2 months.

### Cell viability assay

Firstly, 3000 cells (100 μL) of logarithmic growth cell suspension were inoculated in a 96-well plate and cultivated in an incubator for 24 h. Then, culture medium was replaced with 10% FBS-containing medium with CSE at different concentrations, with or without 100 μM melatonin, and PBS was added to the control cells. Cells were then cultivated in an incubator for 24 h. The medium was then replaced by FBS-free medium containing 10% Cell Counting Kit-8 (CCK-8) reagent (Dojindo, Kumamoto, Japan), and the cells were cultivated in an incubator for 2 h. All experiments were performed in triplicate.

### Western blotting analysis

Total liver protein and HepG2 cells were extracted by radio- immunoprecipitation assay lysis buffer containing protease inhibitors (Beyotime, Shanghai, China) on ice. Protein concentrations were determined using a BCA Protein Assay Kit (Beyotime, Shanghai, China). First, 20 μg of denatured protein was electrophoretically separated by 4–20% sodium dodecyl sulphate-polyacrylamide gel electrophoresis and then transferred onto polyvinylidene fluoride membranes (Millipore, USA). After blocking with 5% skim milk in Tris-buffered saline containing 0.1% tween-20 (TBS-T), the membranes were incubated with primary antibodies against LDLR (1:1000; Proreintech, Wuhan, China) and β-actin (1:2000; Proteintech, Wuhan, China) overnight at 4 °C in a shaker. The membranes were then washed with TBS-T (5 × 5 min) followed by incubation with horseradish peroxidase-conjugated anti-rabbit or anti-mouse IgG (1:5000; Zhongshan Jinqiao Biotechnology, Beijing, China) at room temperature for 1 h. The results were analyzed with Image J software.

### Enzyme-linked immunosorbent assay (ELISA)

Mouse serum concentrations of interleukin-1-beta (IL-1β) (Multi Sciences, Hangzhou, China), interleukin-6 (IL-6) (Multi Sciences, Hangzhou, China) were measured using commercial ELISA kits according to the manufacturer’s instructions and the samples were assessed in triplicate.

### Statistical analysis

All data were presented as mean ± SD. Statistical analysis was performed using GraphPad Prism Version 7.0 software. One-way analysis of variance (ANOVA) was utilized to compare multiple groups. Statistical significance was defined as *p* < 0.05.

## Results

### Neither CS exposure nor melatonin treatment affected mouse body weight

**Fig. 1:**
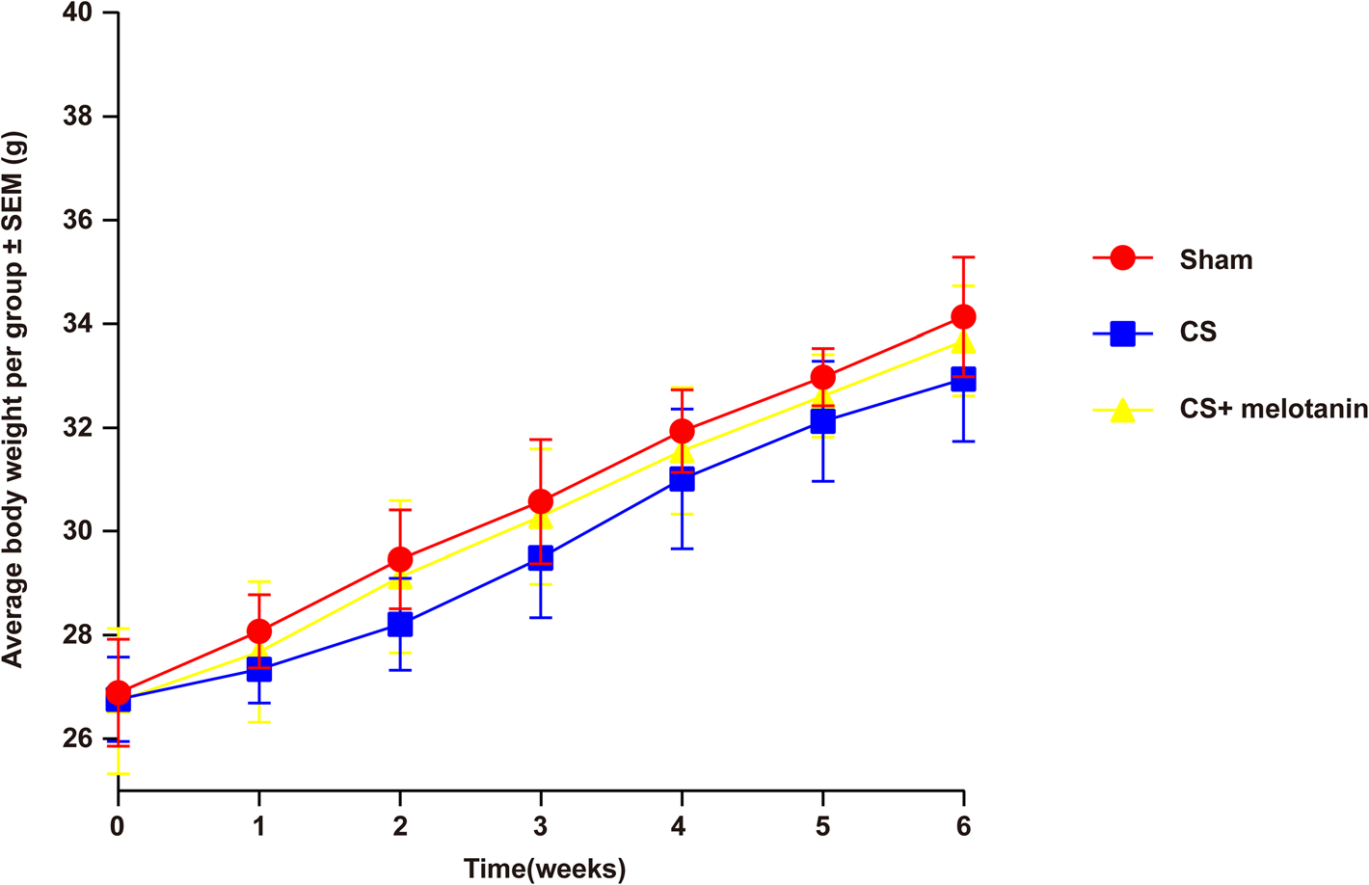
Animal body weight in different groups was measured. CS-exposed mice with and without melatonin treatment grew at the same rate as the sham mice. After the mice were exposed to CS for 6 weeks, there was no significant difference in body weight among the three groups (*P* = 0.8528)

During the 6 weeks of CS exposure and melatonin/saline treatment, the body weights of the mice were recorded every Friday. Results showed that there were no statistical differences among the three groups (Fig. 1).

### Melatonin alleviated the dyslipidemia caused by CS

**Fig. 2:**
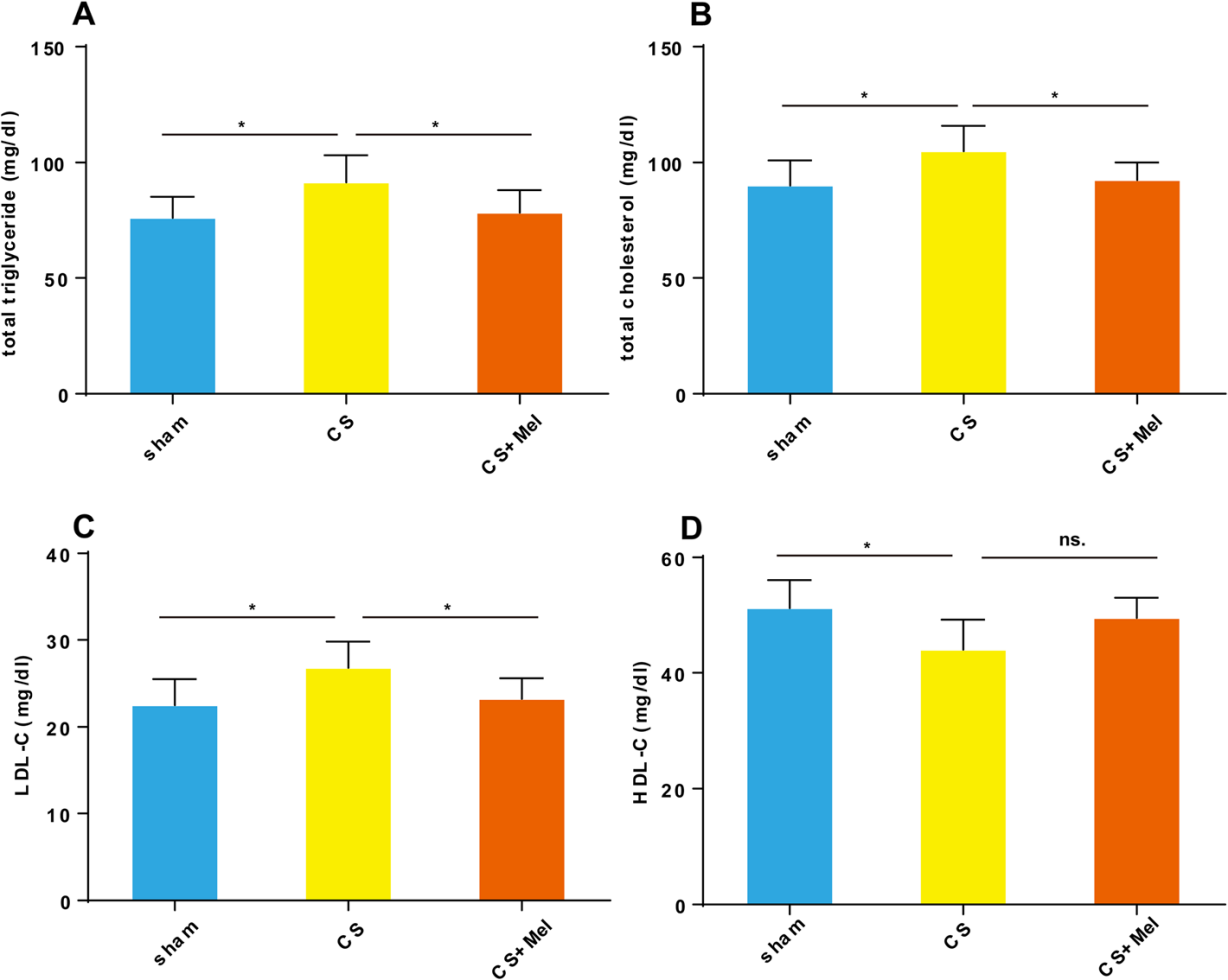
Melatonin alleviated the dyslipidemia caused by CS. The levels of serum TGs, TC, and LDL-C (**a**–**c**) in the CS group were higher than those in the control group, while the HDL-C (**d**) was lower than that in the control group. Furthermore, melatonin supplementation lowered blood lipid levels (**a**–**c**). *P < 0.05, ns. *P* > 0.05

CS exposure increased the levels of serum TGs, TC, and LDL-C by 20.3, 16.7 and 19.0%, respectively, and decreased serum HDL-C levels by 14.1% compared with the control group (*P* < 0.05, Fig. 2a–d), while melatonin treatment significantly improved the CS-affected levels of TGs, TC, and LDL-C (Fig. 2a–c). Although melatonin slightly increased HDL-C level, the difference was not statistically significant (Fig. 2d).

### Melatonin alleviated the increase of inflammatory factors in serum as well as the inflammation and macrophage infiltration in the blood vessel walls caused by CS

**Fig. 3:**
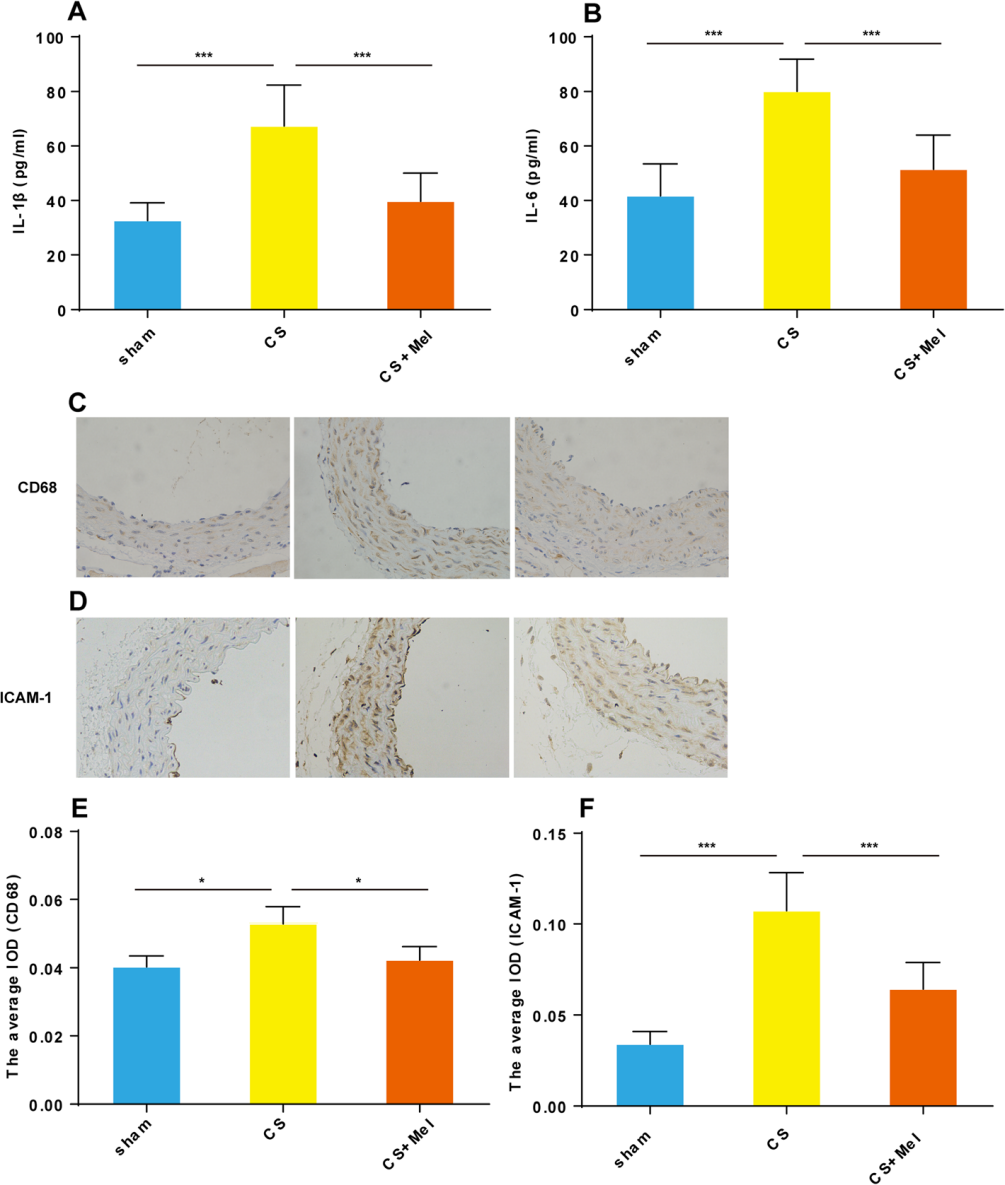
Melatonin alleviated the inflammation caused by CS exposure. The ELISA method was used to detect the expression of IL-1β (**a**) and IL-6 (**b**) in the mouse serum. The thoracic aortas of the mice were stained immunohistochemically for CD68 (**c**) and ICAM-1 (**d**) (200 × magnification). Average integrated optical density (IOD) was used for quantitative analysis (**e**, **f**). *P < 0.05, ***P < 0.001

Macrophages and their secreted cytokines IL-1β and IL-6 play a significant role in promoting atherosclerosis. The serum levels of IL-1β and IL-6 in the three groups of mice were measured by ELISA. Results showed that CS exposure significantly increased the serum levels of IL-1 β and IL-6, while melatonin inhibited this phenomenon (Fig. 3a, b).

IHC was used to detect changes in the expression of CD68 (Fig. 3c) and ICAM-1 (Fig. 3d) in the blood vessel walls. Compared with the sham group, the expression level of ICAM-1, an endothelial injury biomarker, was significantly increased (*P* < 0.001) in the CS-exposed group, but the protein was significantly decreased (*P* < 0.05) by melatonin su*p*plementation (Fig. 3d, f). Inflammation plays a key role in the occurrence and development of atherosclerosis [24]. CD68, an indicator of inflammatory cell infiltration, was highly expressed in mice exposed to CS than in the sham group (Fig. 3c, e). Upon melatonin supplementation, inflammatory cell infiltration in the vascular wall decreased significantly.

### CS and CSE caused a marked decrease in LDLR protein expression in mouse liver and HepG2 cells, which were prevented by melatonin administration

**Fig. 4:**
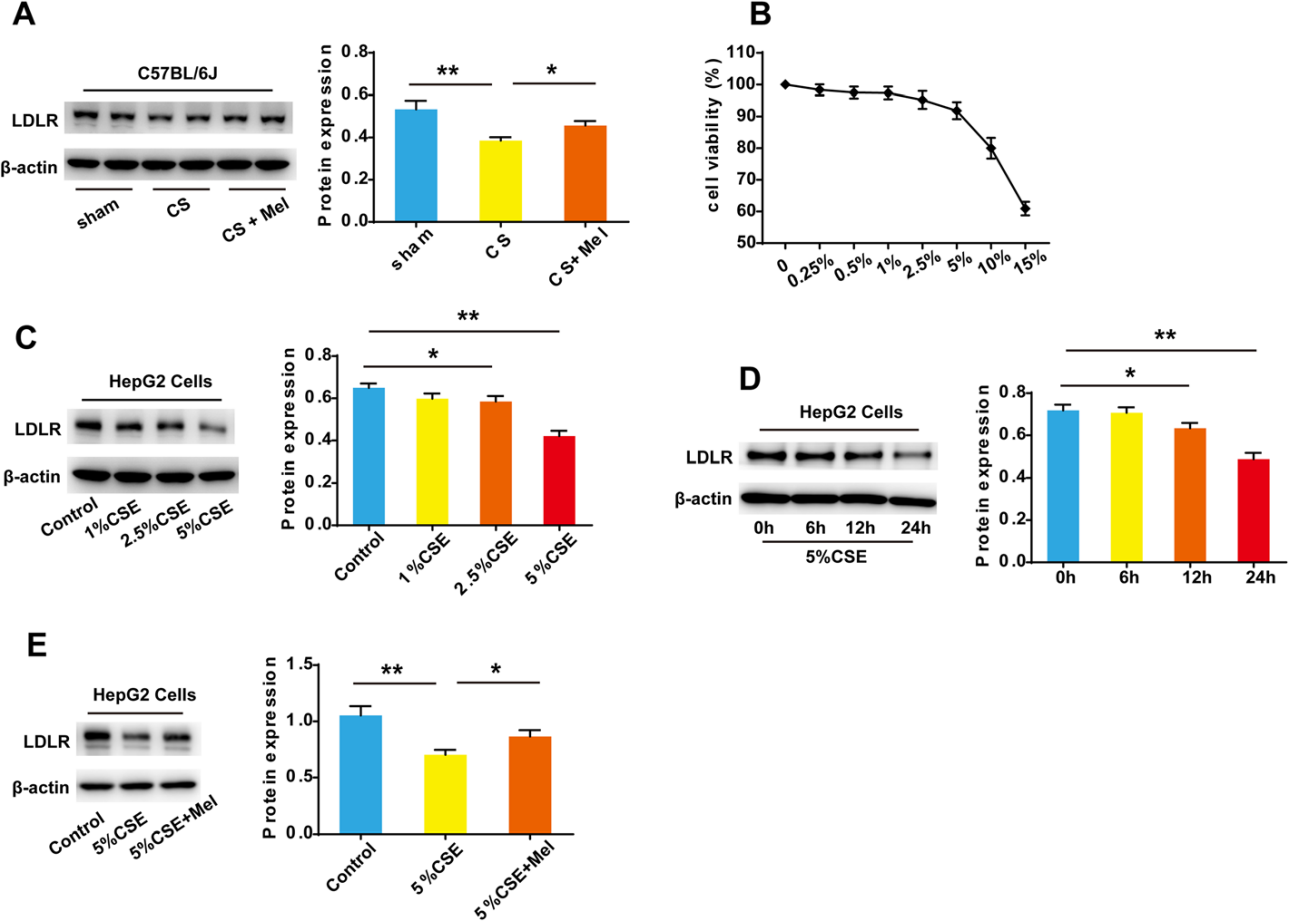
Expression of LDLR protein in mouse liver and HepG2 cells. **a** Expression of LDLR in the liver tissue of the mice in each group. **b** HepG2 cells were stimulated with different concentrations of CSE for 24 h and cell viability was measured using a CCK-8 kit. **c** HepG2 cells were stimulated with CSE at various concentrations (0, 1, 2.5, 5%) for 24 h, and that at 2.5 and 5% CSE markedly inhibited the cellular expression of LDLR protein compared with the control cells. **d** 5% CSE reduced cellular LDLR protein levels in HepG2 cells in a time-dependent manner. **e** Melatonin reversed the downregulation of LDLR protein expression in HepG2 cells induced by CSE. *P < 0.05, **P < 0.001

The expression of LDLR in the livers of the CS-exposed mice was significantly decreased compared with that in the sham group, while the expression level of LDLR protein was increased by melatonin supplementation (Fig. 4a), which indicated that elevated serum LDL-C caused by CS exposure might result from the decreased LDLR protein expression in liver.

To further investigate the effects of CS exposure on LDLR protein expression, CSE was used to stimulate HepG2 cells in vitro. HepG2 cells were propagated in 10% FBS-containing DMEM, and culture medium was changed to 1% FBS-containing DMEM before using CSE to stimulate the cells. The effect of different concentrations of CSE on the viability of HepG2 cells was examined by using a CCK-8 assay kit. When the CSE concentration was 5% or less, the viability of HepG2 cells was above 90%. However, when the CSE concentration was 10% or higher, the cell activity was significantly reduced. Therefore, 5% CSE was used for subsequent experiments. CSE reduced the LDLR protein level in HepG2 cells in a time- and concentration-dependent manner (Fig. 4c, d). However, pretreatment with 100 μM melatonin for 2 h prevented the downregulation of LDLR protein expression induced by CSE treatment in HepG2 cells (Fig. 4e).

## Discussion

CS exposure promotes the development of atherosclerosis through multiple mechanisms and is one of the most preventable risk factors [5]. To some extent, this effect is mainly attributed to the dyslipidemia and inflammatory response of blood vessels [5, 25]. Results in the present study showed that CS exposure caused lipid metabolic disorder and promoted the inflammation of blood vessels, while melatonin supplementation could ameliorate blood lipid disorder and inhibit the inflammation of blood vessel walls.

Passive exposure of C57/6 J mice to CS for 3 h/day for 6 weeks resulted in an increase of LDL-C by 19.0% (*P* < 0.05) and a decrease of serum HDL-C levels by 14.1% (*P* < 0.05) compared with the control group. There is now overwhelming clinical evidence that CS exposure causes lipid metabolic disorder. However, the results of lipid metabolic perturbation caused by active smoking and/or passive smoke exposure are not entirely consistent. For example, Merianos et al. (2018) reported that active CS exposure in adolescents leaded to lower TC, lower HDL-C, and higher TGs levels, while those subjected to passive CS exposure presented lower HDL-C, higher TC, and higher LDL-C levels [26]. However, Bizon et al. (2017) reported that women with active exposure to CS exhibited increased lipid peroxidation and higher TGs, TC, and LDL-C plasma levels along with decreased plasma HDL-C, and the same changes were also observed for female passive smokers [27]. Similarly, the present study showed that the serum levels of TGs, TC, and LDL-C significantly increased after the mice were exposed to CS. Elevated LDL-C is one of the key factors promoting atherosclerosis [2, 6], so lowering LDL-C by statins in individuals produces the regression of atherosclerosis. Compared with statin monotherapy, dual lipid-lowering therapy has more advantages in inducing plaque regression [28].

LDLR protein expressed on the surface of hepatocytes plays a critical role in the regulation of LDL-C levels in the blood [29]. LDLR knockout mice are usually used as models of atherosclerosis, and LDLR genetic mutation is one of the three major causes of familial hypercholesterolemia [30], which is one of the highest risk factors for cardiovascular disease. The molecular mechanism of LDL-C increase caused by smoking is unclear. Therefore, the expression of LDLR protein in the livers of each group was investigated and the results showed that the LDLR protein level of mice exposed to smoke was significantly lower than exposed to fresh air. Furthermore, CSE reduced the LDLR protein level in HepG2 cells in a time- and concentration-dependent manner.

Accumulating evidence supports the critical role of inflammatory mechanisms in atherosclerosis [24, 31, 32]. The atherogenic process starts with the accumulation of LDL-C in the subendothelial site [30, 32]. In the intima, LDL-C is oxidatively modified by reactive oxygen species, taken up by macrophages, and initiates vascular inflammation [31, 33]. Monocytes and other leukocytes as well as proinflammatory cytokines participate pivotally in the various phases of atherosclerosis [24, 34], and CD68 is a marker for macrophages. In the present study, CS exposure increased the infiltration of monocytes/macrophages (CD68 positive cells) into the blood vessel walls and significantly increased the levels of serum IL-1β and IL-6.

ICAM-1, a cell surface glycoprotein, is a member of the immunoglobulin superfamily and is essential for the adhesion and migration of inflammatory cells [35]. As we have previously reported [19], the expression of ICAM-1 was significantly increased upon CS exposure, which implicated that CS exposure might promote the infiltration of inflammatory cells into the vessel walls via ICAM-1. However, melatonin treatment alleviated the increase of ICAM-1 and CD68 caused by CS exposure, suggesting that melatonin could inhibit the inflammation of blood vessel walls by inhibiting the expression of ICAM-1 and the migration of CD68 positive cells to the vessel walls.

A growing body of clinical data has revealed that melatonin could improve dyslipidemia and reduce the risk of cardiovascular events in patients with hyperlipidemia [16, 17]. Melatonin can also improve lipid dysmetabolism in high-fat-diet-fed mice by reprogramming gut microbiota [36]. However, the effects of melatonin on lipid metabolic disorder caused by CS exposure have not been reported. These results showed that supplementation with 10 mg/kg/d melatonin significantly improved the CS-impaired levels of TGs, TC, and LDL-C. The expression of LDLR in the livers of CS-exposed mice was reduced, which could be restored by melatonin treatment.

To validate these findings, in vitro experiments using HepG2 cells were performed and the results showed that treatment with 100 μM melatonin alleviated the decrease in LDLR expression in HepG2 cells stimulated with CSE. These data suggest that melatonin may regulate CS-induced dyslipidemia by upregulating the expression of LDLR on the surfaces of hepatocytes.

## Conclusions

In conclusion, CS exposure leads to lipid metabolic disorder and inflammation of blood vessel walls in C57BL/6 J mice. Melatonin prevents CS-induced dyslipidemia and inflammation, and this may have important implications for the prevention and treatment of atherosclerosis, especially that caused by smoking. However, the specific mechanism of CSE leading to the decrease of LDLR expression in hepatocytes needs to be further investigated.

## Data Availability

Data in the current study are available from the corresponding author on reasonable request.

## Abbreviations

CS: Cigarette smoke
LDL-C: Low-density lipoprotein cholesterol
LDLR: Low-density lipoprotein receptor
HDL-C: High-density lipoprotein cholesterol
TC: Total cholesterol
TGs: Triglycerides
CSE: Cigarette smoke extract
SPF: Special pathogen free
PUMCH: Peking Union Medical College Hospital
ICAM-1: Intercellular adhesion molecule-1
CD68: Cluster determinant 68
PBS: Phosphate-buffered saline
DMEM: Dulbecco’s modified Eagle’s medium
IL-1β: Interleukin-1-beta
IL-6: Interleukin-6
IOD: Integrated optical density
CCK-8: Cell Counting Kit-8
TBS-T: Tris-buffered saline containing 0.1% tween-20
ELISA: Enzyme-linked immunosorbent assay
